# Evaluation of efficacy of a new hybrid fusion device: a randomized, two-centre controlled trial

**DOI:** 10.1186/1471-2474-15-294

**Published:** 2014-09-05

**Authors:** Jan Siewe, Jan Bredow, Johannes Oppermann, Timmo Koy, Stefan Delank, Peter Knoell, Peer Eysel, Rolf Sobottke, Kourosh Zarghooni, Marc Röllinghoff

**Affiliations:** Department of Orthopaedic and Trauma Surgery, University of Cologne, Kerpener Strasse 62, 50924 Cologne, Germany; Department of Orthopaedic and Trauma Surgery, University of Halle/Saale, Magdeburger Strasse 22, 06112 Halle, Germany; Department of Orthopaedic Surgery, Medical Center “StädteRegion Aachen GmbH”, Mauerfeldchen 25, 52146 Würselen, Germany

## Abstract

**Background:**

The 360° fusion of lumbar segments is a common and well-researched therapy to treat various diseases of the spine. But it changes the biomechanics of the spine and may cause adjacent segment disease (ASD). Among the many techniques developed to avoid this complication, one appears promising. It combines a rigid fusion with a flexible pedicle screw system (hybrid instrumentation, “topping off”). However, its clinical significance is still uncertain due to the lack of conclusive data.

**Methods/Design:**

The study is a randomized, therapy-controlled, two-centre trial conducted in a clinical setting at two university hospitals. If they meet the criteria, outpatients presenting with degenerative disc disease, facet joint arthrosis or spondylolisthesis will be included in the study and randomized into two groups: a control group undergoing conventional fusion surgery (PLIF - posterior lumbar intervertebral fusion), and an intervention group undergoing fusion surgery using a new flexible pedicle screw system (PLIF + “topping off”), which was brought on the market in 2013.

Follow-up examination will take place immediately after surgery, after 6 weeks and after 6, 12, 24 and 36 months. An ongoing assessment will be performed every year.

Outcome measurements will include quality of life and pain assessments using validated questionnaires (ODI - Ostwestry Disability Index, SF-36™ - Short Form Health Survey 36, COMI - Core Outcome Measure Index). In addition, clinical and radiologic ASD, sagittal balance parameters and duration of work disability will be assessed. Inpatient and 6-month mortality, surgery-related data (e.g., intraoperative complications, blood loss, length of incision, surgical duration), postoperative complications (e.g. implant failure), adverse events, and serious adverse events will be monitored and documented throughout the study.

**Discussion:**

New hybrid “topping off” systems might improve the outcome of lumbar spine fusion. But to date, there is a serious lack of and a great need of convincing data on safety or efficacy, including benefits and harms to the patients, of these systems. Health care providers are particularly interested in such data as these implants are much more expensive than conventional implants. In such a case, randomized clinical trials are the best way to evaluate benefits and risks.

**Trial registration:**

NCT01852526

## Background

Lumbar spine fusion is a controversial surgery. While some evidence suggests that it could be more efficient than commonly used nonsurgical treatment, there is also evidence to the contrary [1]. But in spite of the inconclusive results, it remains a well-established and frequently used surgical treatment for various diseases of the lumbar spine and chronic low back pain.

Spinal fusion is known to change the biomechanics of the spine, e.g. it can result in increased motion in the segments adjacent to the fused level. Outweighing the initial clinical benefits from the surgery, this motion may cause adjacent segment disease (ASD) that is defined as the clinical presence of symptoms which correlates to degenerative disease adjacent to the index level with the radiographic presence of disease. It must be distinguished from adjacent segment degeneration, a radiological term that does not lead to clinical symptoms [2].

Thus different techniques (disc arthroplasty and dynamic stabilization) have been developed to prevent this degeneration [3]. In this context, the posterior dynamic stabilization (PDS) is one of the most rapidly evolving technique, including various types of flexible pedicle screw systems, such as the “topping off” version mentioned above. Other PDS devices include interspinous spacers used by some surgeons together with a monosegmental rigid fusion [4]. To simplify the discussion of these devices, Khoueir et al. [5] classified them into:

1. Interspinous spacer devices;
2. Pedicle screw/rod-based devices; and
3. Total facet replacement systems.

The concept behind these devices is to maintain or restore intervertebral motion in a controlled manner, whether by preventing excessive spinal movement or by dampening the kinetic energy. They are designed to mimic the behaviour of the healthy spinal column [5, 6]. Various types of flexible pedicle screw systems (e.g. Dynesis™, DSS™, cosmicMIA™) and “topping off” systems (e.g. DTO™, BalanC™,) have been developed.

The ”topping off” technique has been reported to provide dynamic stabilization and to reduce degeneration in the adjacent segment [7, 8]. Some studies report better clinical outcomes in patients with partial fusion than with solid fusion, they suggest that reducing, rather than eliminating, segmental motion alleviates pain. It could be a promising alternative to multilevel fusion [9] and, according to Coe et al., to rigid fusion [10].

One of the most extensively used posterior dynamic stabilization systems is Dynesys®, in which pedicle screws are connected across spinal motion segments with non-elastic bands to provide controlled motion. In early clinical outcomes, it has been shown to lessen pain and disability in patients with degenerative spondylolisthesis and central or lateral spinal canal stenosis. Comparable clinical outcomes of other pedicle-based dynamic stabilization systems have also been reported [11, 12]. However, in a prospective, randomized clinical trial of 60 patients comparing hybrid with conventional fusion over 6 years of follow-up, clinical results did not differ between the groups (ODI, VAS, satisfaction). Hybrid fixation caused less degeneration in the adjacent segment, but showed a higher rate of implant failure. This study group did not recommend prophylactic dynamic stabilization [13].

In patients with lumbar spinal stenosis that underwent decompression and stabilization with the Accuflex™ dynamic system, Reyes-Sánchez et al. report a relatively high hardware failure (22%), but also clinical benefits and cessation of the degenerative process in 83% of the patients [11].

As a summary, it can be said that there is still no evidence of any clinical benefit from these systems.

Another issue is whether the radiographically detectable adjacent degeneration is an indicator of clinical outcome. As already mentioned, spinal fusion creates increased strain on adjacent levels and can result in ASD. Adjacent instability has been reported even 6 to 12 months after surgery [3, 14], with varying average rates. In a retrospective study, Cheh et al. identified radiographic ASD in 42.6% of the patients (average follow-up of 7.8 years). Oswestry scores were worse in patients with radiographic ASD. Clinical ASD developed in 43% [3]. Other authors reported an incidence of ASD of up to 24% (average follow-up of 39 months). In that study, instability developed more frequently above the fusion [14]. Yang et al. found a significant correlation between clinical outcome and ASD [15]. In a 30-year follow-up comparing patients undergoing various spinal procedures (fusion, discectomy, decompression), Kumar et al. [16] found that the incidence of radiographic changes in levels above the operated region was twice higher after fusion than after the other procedures. In contrast, validated scales and functional testing (e.g. SF-36) showed no significant differences in outcome. The authors concluded that radiographic changes do not necessarily lead to functional impairment in all patients. Other evidence suggests that radiologic degeneration of the superior adjacent segment does not correlate with clinical results [17]. Ghiselli et al. reported an adjacent segment disease rate of 16.5% after 5 years in a collective of 215 patients who had undergone lumbar spine arthrodesis. This rate went up to 30.1% at ten years follow-up [18]. However, a previous review mentions a correlation between fusion and adjacent segment degeneration compared to arthroplasty. This correlation appears to be even stronger if adjacent segment disease is observed, thus underlining the impact of fusion on the adjacent segments [2]. In this context, sagittal imbalance is another factor of considerable importance; some studies indicate that it favours the development of the disease [19, 20].

### Objective

The objective of the study is to address the following questions:

Does posterior hybrid stabilization with a new topping off device lead to a better or at least equivalent clinical outcome compared to standard fusion? Clinical outcome assessment will be performed using ODI, SF-36 and COMI questionnaires.

Does it prevent adjacent instability/segment disease?

Does radiographic ASD correlate with clinical outcome?

Does this new device lead to a higher complication rate (e.g. implant failure) than standard fusion?

## Methods and design

The study is a randomized, parallel-group, therapy-controlled trial in a clinical setting at two university hospitals. Outpatients presenting with degenerative disc disease, symptomatic facet joint arthrosis or spondylolisthesis will be assessed for inclusion. They will be asked to give an informed consent and will be randomized before surgery. Follow-up examinations will take place immediately after surgery, and after 6 weeks, 3 months and 6 months, the total duration of the study. Furthermore data will be assessed after 12, 24 and 36 months for a supplemental investigation. A further assessment will be performed every year, as it may be impossible to pronounce on ASD after 36 months.

This trial has received the approval of the ethics committee of the medical faculty of the University of Cologne under the reference number 12–231. The Clinical Trial Centre of the University of Cologne will monitor and manage the data. The research will be carried out in compliance with the Helsinki Declaration [21].

### Participants and recruitment

Outpatients over 30 years of age presenting with degenerative disc disease, facet joint arthrosis or spondylolisthesis, and indications for monosegmental lumbar spine fusion will be eligible for trial inclusion. Radiologic inclusion criteria are summarized in the Appendix.

The presence of radiologic degeneration of the adjacent segment (Pfirrmann grades II-IV in MRI findings [22]) without signs of instability will be a condition for inclusion. The definition of radiologic and clinical instability and the other inclusion criteria are summarized in the Appendix.

Experienced spine surgeons will approach and recruit the study subjects. The number of patients who undergo primary monosegmental fusion in the departments is estimated at 200 per year and the number of recruited patients at 20 per year per centre.

Patients participating in parallel interventional studies as well as patients with lumbar scoliosis (>25° cobb angle), spondylolisthesis Meyerding IV, adjacent segment instability and/or radiologic ASD worse than Fujiwara grade II or Pfirrmann grade IV [22, 23] will be excluded from this study. This also applies to patients with indication for multilevel fusion. The other exclusion criteria are summarized in the Appendix.

### Interventions

#### Patients will receive one of two treatments

- Monosegmental posterior lumbar intervertebral fusion (PLIF)
- Hybrid system (PLIF + flexible pedicle screw system above the fusion)

#### Control group - conventional PLIF

The control group will be treated with a conventional, monosegmental posterior lumbar spine fusion with a rigid pedicle-based instrumentation and posterior intervertebral cages (PLIF), the current well-established therapy for several pathologies of the lumbar spine (see Appendix under “Inclusion Criteria”). Thus, the control group will receive the standard of care. This is the only acceptable control/comparison in a trial of this kind. Surgery will be performed using the following devices: S^4^® AESCULAP AG, Cage: Wave® Cage (Fa. Medtronic®).

#### Intervention group - hybrid system

The intervention group will be treated with a hybrid system with PLIF (rigid pedicle-based instrumentation and posterior intervertebral cages) and a flexible pedicle screw system above the fusion. Surgery will be performed using the following devices: S^4^® Dynamic rod (AESCULAP AG, Tuttlingen), Wave® Cage, (Fa. Medtronic ®). The dynamic connecting rod of the S4 Spinal System is used for the dynamic monosegmental stabilization through the dorsal approach and the fusion of one or two adjacent segments. It consists of a spring element, a short rod section on one side, and another rod section that varies in length on the other side. The materials used in the implant are pure titanium and titanium forged alloy Ti6Al4V. The prototype of this dynamic implant has already been biomechanically tested and compared with other dynamic devices and a rigid one; no significant ROM reduction (extension, flexion, lateral bending) was detected. In addition, the implant brought about a significant reduction of axial rotation [24].

### Surgery and treatment

Only skilled spine surgeons (experience of at least 30 fusions-procedures/per year) will participate in the trial. To avoid variations in surgical procedure (e.g. size of decompression/approach), intraoperative photos and an instructional video on the standards of the procedure will be provided to the surgeons. In both groups, autologous bone spongiosa, harvested during the decompression procedure, will be implanted in the intervertebral disc space with the intervertebral cages.

The patients will have the surgical drain removed 2 days after surgery. Beginning on the day after surgery, both groups will receive physical therapy. Patients will be discharged only after appropriate recovery if wound healing is progressing normally. They will remain in the hospital for 8–10 days. After hospital discharge, they will continue to receive physical therapy as outpatients, but not in standardized form to reproduce real conditions.

### Outcome measures and assessments

#### Primary outcome measures

This investigation focusses on the subjective and objective clinical benefits for the patient. The ODI scores will be used to evaluate the functional outcomes after 6 weeks and after 6, 12, 24 and 36 months after surgery. The study hypothesis is:

The improvement in ODI scores from baseline to 6 months after surgery is clinically superior in the group with D-Rod (μRod) compared with the group without D-rod (μ).

To prove it, the following null hypothesis must be rejected: H0: |μRod - μ| ≤ MCID;

The alternative hypothesis is: H1: |μRod - μ| > MCID, with MCID assigning for minimum clinically important difference.

The hypothesis will be tested using a two-sided t-test for independent samples with a significance level of 5%.

In accordance with the recommendations of the European Agency for the Evaluation of Medicinal Products (EMEA), the patient’s bodily function may not be impaired if the only medical benefit is to relieve pain [25].

The ODI (cross-cultural adaption of the ODI version 2.1 for use with German-speaking patients) is a standardized, condition-specific questionnaire that gives a subjective percentage score of level of function (disability) in 10 everyday activities of daily living to be completed by patients rehabilitating from low back pain [25].

In the past, spine surgery investigations have focused on technical outcomes; now the main interest has turned to clinical outcomes [26, 27], measured by the ODI and SF-36™ , as in recent important trials (RCTs) [28, 29]. Radiologic findings are poor indicators of clinical outcome as it is unclear whether they correlate with clinical success.

### Secondary outcome measures

1. PCS of the SF-36™. The SF-36™ is the most frequently used generic health status measure worldwide that includes parameters such as pain and walking distance. It has been further developed to comprise physical (PCS) and mental health (MCS) component scores, thus easing the interpretation and cross-cultural comparison. It is a standardized questionnaire used to evaluate the patients’ health-related quality of life. It yields an 8-scale profile of functional health and well-being scores, as well as psychometrically based physical and mental health summary measures, and a preference-based health utility index. Thus, the SF-36™ is a good measure of primary endpoint. The patients' answers are scored and added together to yield a sum total. The SF-36™ PCS scores of the experimental and control groups will be compared to further quantify patient outcomes. In the subsequent follow-ups, the SF-36™ will be repeated to detect any clinical impairment and/or clinical or radiologic instability. This follow-up will continue for up to 3 years and then each year after surgery. In cases of impairment, X-ray is indicated at any point of time. In total, MCS and 8 individual dimensions and subscales of the SF-36™ (version 4.0) are used to confirm and clarify the primary outcome results.
2. COMI questionnaire (2008 version) - a short, patient-oriented, multidimensional outcome instrument validated for patients with spinal disorders.
3. X-rays will be taken after 6 weeks, and after 6, 12, 24, and 36 months. A further assessment will be performed every year. These are common intervals after surgical implantation without extraneous radiation. If adjacent instability is discovered on a X-ray, the clinical outcome will be reevaluated. The criteria for both radiologic ASD and adjacent instability are listed in the Appendix (definition of radiologic instability and Weiner’s classification). Experienced radiologists will evaluate the pre- and postoperative X-rays to calculate the score in Weiner’s classification. Furthermore, after 6 months, full-length X-rays of the spine will be taken to assess sagittal balance parameters (pelvic incidence, sacral slope, pelvic tilt, lumbar lordosis, C7 plum line, SVA). At each follow-up visit, bone fusion will be evaluated by indirect signs (screw loosening, implant failure, loss of reduction).
4. Clinical ASD will be assessed (see definition in the Appendix).
5. Work disability at the time of and after surgery, and duration until return to work. At each visit, patients will be asked to grade their disability, and whether or when they have returned to work.
6. Inpatient mortality and mortality at 6 months.
7. Surgery-related data (blood loss, length of incision, duration of surgery) will be taken from the surgical report.
8. During the study, type and dose of pain medication administered to the patients will be documented. If the patient complaints of pain, the medication will be adjusted. The adjustment will be recorded on the CRF.
9. Intraoperative and postoperative complication rate (e.g. implant failure- see Appendix under “Safety”)
10. Adverse Events (AE) and Serious Sdverse Svents (SAE) will be monitored and documented throughout the study (see Appendix under “Safety” and “AE and SAE”).

### Sample size

On the basis of the data provided by Parker et al., the standard deviation is set at 11.3 points and the minimal clinically relevant difference at 11 for the changes in the Oswestry Disability Index (ODI) scores between the baseline and six months. In order to detect this clinically relevant difference between the two treatment groups, using a two-sided t-test with a significance level of 5% and a power of 80%, a total of 36 patients is required (18 per group) [30].

Assuming a drop-out rate of 10% until follow-up, 40 patients must be recruited for the study.

At a recruitment rate of 50% of the eligible patients and a rate of 60% of eligible patients in the screened population, 80 patients must be eligible among the 133 screened patients.

At a screening rate of approx. 100 patients in 12 months, duration of recruitment would be 16 months. To comply with the CONSORT requirements, we will provide a flow diagram of the participants. For each group, it will show the numbers of participants who were randomly assigned, received intended treatment, and were analysed for the primary outcome. Furthermore a diagram will illustrate for each group, losses and exclusions after randomization, together with reasons [31].

### Randomization

The patients will be assigned to the two treatment groups using an electronic randomization system provided by Aesculap®.

Balanced centre-stratified randomization with blocks of variable length. The randomization algorithm is implemented using statistical package SAS 9.2 and modified according to Dmitrienko et al. [32].

### Data management

At the study sites, the data will be entered online in a validated study database (eCRF) by trained personal. During data entry, plausibility tests will be carried out to tackle inconsistencies immediately. Later, a data manager will repeatedly check the data for completeness and plausibility and resolve the inconsistencies in concert with the centre. The central IT infrastructure will be provided by the ZKS.

The study database will be running on validated study software (MACRO). The study database itself will be validated before the data are entered. It will store all changes made to the data in an audit trail with a user and role concept adaptable to the specific study. The database will be integrated in a general IT infrastructure and security architecture with firewall and backup system. The data will be backed up daily.

### Data analysis

#### Primary target variable

The primary target variable is the functional change in the lumbar spine between baseline and 6 months as measured by the Oswestry Disability Index (ODI). The two-sided t-test for independent samples will be used as confirmatory test to compare the intervention group with the control group.

Analysis will be based on the intention-to-treat (ITT) principle. Consistency of the results will be verified by additional per-protocol (PP) subgroup analysis.

In addition, the results will be evaluated, focusing separately on each of the three diseases: degenerative disc disease, degenerative spondylolisthesis und spondylolisthesis. This applies to both the results from the ODI and to all other secondary target variables.

#### Secondary target variables

All secondary target variables, such as frequency, average and standard deviation will be tabulated per point of time (baseline, 6 weeks, 6 months) and per treatment arm. The historical data for ODI, MCS of SF-36 of PCS of SF-36 will be expressed as AUCs (areas under the curve) adjusted for the observation period. Once the data have been collected after 6 weeks, 6 months and 36 months, the missing values, if any, will be interpolated after 12 or 24 months, or the AUC will not be evaluated. The two-sided t-test for independent samples will be used to compare the intervention group with the control group. The Logrank test will be used to compare the treatment groups in terms of survival time to instability measured by the Kaplan-Meier method. The link between time to instability and the ODI will be investigated by correlation analysis. Following evaluation of the primary endpoint, the ITT population and the PP population – as sensibility analysis – will be analysed.

#### Monitoring

To ensure proper conduct of the study and reliability of the results, clinical monitors will visit the trial centres.

For one patient at each trial centre, 100% source data verification will be carried out.

For some randomly chosen patients, source data from the questionnaires will be partially checked against the case report form data. For each patient, the presence of a written consent will be verified.

### Biases

To avoid potential biases, this study is planned as randomized trial. Blinding will not be attempted because it cannot be successfully implemented as X-rays must be taken. Since the hybrid implant is expected to bring benefits in comparison with the conventional one, it must be tested in comparison to this control group.

To avoid bias without blinding design, the following measures are planned:

1. Different trial physicians will carry out the surgery and the questionnaire survey
2. Different trial physicians will carry out the questionnaire survey and the evaluation of the X-rays
3. The patient will be instructed not to give any details of his treatment to the trial physician
4. The trial physicians will be trained to evaluate the X-rays
5. Intraoperative photos will be provided to compare the surgical techniques (approach, size of decompression) and, if need be, a video of the surgery for standardisation and training purposes

### Adverse and serious adverse events

#### Adverse event

An Adverse Event is any untoward medical occurrence in a study patient that may or may not have a causal relationship with the study treatment. Abnormal laboratory test values will be recorded as AE only if they require treatment.

##### Concomitant diseases

In this context, deterioration of a pre-existing disease is also to be regarded as an Adverse Event. But it will not be regarded as an Adverse Event if it is due to a treatment that was already planned before the patient was enrolled in the study.

##### Pregnancy

In this study, the occurrence of pregnancy is considered as an Adverse Event. Before surgery, laboratory tests will be conducted to exclude pregnancy in women below 50 years of age.

##### Laboratory test values

Any abnormal laboratory test values during the study will be checked for plausibility and evaluated for clinical relevance by the responsible investigator. If during the study, an abnormal test value is found that, in the baseline visit, was not considered to be clinically relevant, but is now considered to be clinically relevant, it must be recorded as an AE in the eCRF.

### Serious adverse event

The present clinical study is not subject to the Sections 20-23a of the German Medical Product Law (Medizinproduktgesetz – MPG), but its definitions will apply here. A Serious Adverse Event is any untoward occurrence in a clinical trial or in a performance evaluation text subject to approval that

- directly or indirectly leads to death; or
- have led, could have led, or could lead to a serious deterioration in the state of health of a study subject, of a user or of another person, irrespective of whether the occurrence has been caused by the medical product.

This definition of a SAE will serve as basis for the documentation of Serious Adverse Events during the study.

### Ascertainment of AE and SAE

The study centre concerned is responsible for recording and reporting AEs and SAEs. The study director/main investigator will ensure that all persons involved in the treatment of the study patients are fully aware of their responsibilities in the event of Adverse Events. At each visit, the patients will be asked if they have experienced any Adverse/Serious Events. The Adverse Events will be recorded both in the patients’ medical record and in the survey questionnaires. At each visit, the physician will review the patients’ medical records to determine whether Adverse Events have occurred.

If an Adverse Event occurs, the patient in question, irrespective of the causal relationship between the Adverse Event and the study treatment, must be kept under constant supervision. In any case, the patient must remain under observation until the symptoms have subsided, or the laboratory test values have returned to acceptable levels, or a plausible explanation has been found for the Adverse Event, or until the patient has died, or has been discharged from the study (last visit after 6 months or 36 months).

All Adverse Events will be recorded in the CFR, including the following information:

- Time and date of start and end ,
- Gravity ,
- Relationship to the study therapy
- Serious or not serious ,
- Expected or not expected ,

## Discussion

New “topping off” hybrid systems might reduce the complications associated with lumbar spine fusion. But to date, there is no convincing evidence to confirm this hypothesis. This study is intended to fill this gap by providing highly reliable data and a preliminary evaluation of the advantages and disadvantages of a device that was launched in 2013. These systems are much more expensive than conventional implants, they should not be implanted if they do not bring any appreciable benefit, an aspect which is of interest to the health care providers. This study is designed as a randomized clinical trial, a format highly recommended to evaluate the benefits and risks to patients of new devices.

In the intervention group, the approach to the spine will be larger than in the fusion group. This in itself could bring about a negative outcome compared to that of standard fusion (e.g., due to muscle damage, blood loss, higher risk of infection). But implant failure rates can still be compared between the groups. The crucial question, however, is the ability to prevent ASD.

To use a placebo in the control group would be fundamentally unethical. Not only would it deprive the patient of adequate treatment, but would also require an invasive procedure without implantation, thus exposing him/her to significant risks without benefit. Furthermore, there is no “placebo device” that could be used here.

## Appendix

### Inclusion criteria

- Informed consent
- Legal capacity
- Age ≥ 30 years
- Indication for monosegmental lumbar spine fusion L2-S1 with osteochondrosis Modic grades I-III
- Indication for monosegmental lumbar spine fusion L2-S1 with spondylolisthesis Meyerding grades I-III (signs of instability in the functional x-rays and a.p. and lateral view; see definition of radiologic instability).
- Indication for monosegmental lumbar spine fusion L2-S1 with symptomatic facet joint arthrosis or degenerative disc disease (>Fujiwara grade II [23] or > Pfirrmann grade IV [22])
- Radiologic signs of degeneration in the adjacent segment of the intended fusion without signs of instability
- Definition of adjacent segment degeneration (MRI)
  - Pfirrmann [22] grades II-IV
  - Grade I: Homogeneous bright white structure
  - Grade II: Inhomogeneous white structure, possible horizontal bands
  - Grade III: Clear distinction between annulus and nucleus
  - Grade IV: Non-collapsed disk space
  - Grade V: Collapsed disk space
- Definition of radiologic instability (x-ray: a.p. and lateral view, extension and flexion):
  1. Spondylolisthesis >4 mm (anterior or posterior translation) [3]
  2. Segmental kyphosis >10° [3]
  3. Rotatory hypermobility >15° [14]
  4. Complete collapse of the disc [3]
  5. Lateral translation >3 mm [14]
  6. Disc wedging >5° [14]
  7. Deterioration in Weiner’s classification of 2 or more grades in the follow-up evaluation [1, 27]

### Weiner’s classification

0 = No disease, defined by normal disc height, no spur formation, no eburnation, and no gas

1 = Mild disease, defined by <25% disc space narrowing, small spur formation, minimal eburnation, and no gas

2 = Moderate disease, defined by 25%-75% disc space narrowing, moderate spur formation, moderate eburnation, and no gas

3 = advanced disease, defined by >75% disc space narrowing, large spur formation, marked eburnation, gas present

### Exclusion criteria

- Motor deficit
- Cauda equina syndrome
- Previous surgical intervention of the lumbar spine
- Relevant peripheral neuropathy
- Acute denervation subsequent to radiculopathy
- Scoliosis with Cobb angle greater than 25°
- Spondylolisthesis > Meyerding grade III
  - Radiologic signs of degeneration in the adjacent segment of the intended fusion with signs of instability (for definition, see inclusion criteria)
  - No radiologic signs of degeneration in the adjacent segment of the intended fusion (for definition, see inclusion criteria)
  - Radiologic signs of degeneration in the adjacent segment of the intended fusion with > Fujiwara grade II [23] or > Pfirrmann grade IV [22]
  - Signs of instability in any lumbar spine segment other than that undergoing fusion (patients with indication for multilevel fusion are excluded)
- General contraindication for elective lumbar spine surgery
- Pathologic fracture
- Osteoporosis with pathologic fracture
- Active systemic infection
- Rheumatic disease
- Disease of bone metabolism (e.g. Paget’s Disease)
- Bone metastasis
- Local infection of lumbar spine
- Seizure disorder
- Chronic ischemia according to Fontaine classification IIb-IV
- Severe heart insufficiency (NYHA III-IV)
- Blood coagulation disorder or blood thinning therapy
- Cortisone intake more than one month in the last 12 months before randomization
  - Simultaneous participation in another clinical trial in the 30 days before randomization
- Known allergy or intolerance to the implants
- Dependency on investigator
- Lack of familiarity with the German language
- Placement in an institution by governmental or juridical order
- Absent legal capacity
- Pregnancy
- Lactation period

### Definition of clinical ASD [14]

1. Symptomatic spinal stenosis
2. Mechanical back pain
3. Sagittal or coronal imbalance

### Safety

#### Implant failure

1. Screw loosening
2. Screw breakage
3. Rigid rod breakage
4. Dynamic rod breakage

#### Potential complications

- Bleeding/secondary bleeding
- Wound infection
- Epidural infection/abscesses
- Meningitis
- Vertebral fracture
- Dislocation/fracture/implant migration
- Implant malposition
- Foreign-body reaction
- Complications in anaesthesia
- Paralyses, neurologic deficit
- Dura leakage
- Sensibility disorders
- Necessity of a revision
- Thrombosis/pulmonary embolism
- Allergic reaction following administration of antibiotics
- Risk of HIV, hepatitis B, C etc. after blood transfusion

## Abbreviations

a.p.: Anterior-posterior
ASD: Adjacent segment degeneration/disease
COMI: Core outcome measure index
MCS: Mental health component score
MRI: Magnetic resonance imaging
NYHA: New York heart association
ODI: Oswestry disability index
PCS: Physical health component score
PLIF: Posterior lumbar intervertebral fusion
QALY: Quality-adjusted life years
RCT: Randomized clinical trial
SF-36™: Short form health survey-36
VAS: Visual analogue scale.
